# The Impact of an MDP-Containing Primer on the Properties of Zinc Oxide Networks Infiltrated with BisGMA-TEGDMA and UDMA-TEGDMA Polymers

**DOI:** 10.3390/ma18010137

**Published:** 2024-12-31

**Authors:** Benjamin Wellhäußer, Lena Marie Saure, Fabian Schütt, Franziska Scherer, Sebastian Wille, Matthias Kern

**Affiliations:** 1 Department of Prosthodontics, Propaedeutics and Dental Materials, School of Dentistry, Christian-Albrechts University at Kiel, Arnold-Heller-Straße 16, 24105 Kiel, Germanyswille@proth.uni-kiel.de (S.W.); mkern@proth.uni-kiel.de (M.K.); 2 Department for Materials Science, Faculty of Engineering, Christian-Albrechts University at Kiel, Kaiserstraße 2, 24143 Kiel, Germany; lms@tf.uni-kiel.de (L.M.S.); fas@tf.uni-kiel.de (F.S.)

**Keywords:** zinc oxide, polymer-infiltrated ceramic network, biaxial flexural strength, water absorption, hardness, roughness

## Abstract

This study was conducted to evaluate the material properties of polymer-infiltrated zinc oxide networks (PICN) and the effect of using a phosphate monomer-containing primer applied before polymer infiltration. A total of 148 ZnO-network (zinc oxide) specimens were produced: *n* = 74 were treated with a primer before polymer infiltration and light curing, while the remaining specimens were untreated. Each group was divided into two subgroups (*n* = 37) based on the infiltrating polymer: UDMA (aliphatic urethane-dimethacrylates)-TEGDMA (triethylene glycol-dimethacrylate) or BisGMA (bisphenol A-glycidyl-methacrylate)-TEGDMA. Additionally, *n* = 7 specimens of each polymer type were prepared for comparison. Then, biaxial flexural strength was measured before and after 150 days of water storage at 37 °C, including 37,500 thermal cycles (5 °C to 55 °C). The Vickers hardness, surface roughness, and water absorption at 37 °C were also tested. The initial biaxial flexural strength was reduced in the ZnO network specimens compared to in the pure polymers. Primer application improved the flexural strength, though the strength of BisGMA-TEGDMA significantly decreased after water storage. The ZnO network increased hardness, and the polymer-infiltrated networks showed higher roughness post-grinding and absorbed less water than the pure polymer groups. The ZnO networks did not improve the flexural strength over that of the pure polymers. However, the primer’s positive impact and the network’s long-term stability suggest potential if the network structure can be modified to contain thicker, more stable branches.

## 1. Introduction

Conventional dental composites usually have two main phases: an inorganic phase, consisting of filler particles varying in size from submicro- to macro-fillers, and an organic phase [1], with polymers featuring one, two, or three terminal double bonds. The monomers used are either aromatic dimethacrylates or aliphatic urethane dimethacrylates (UDMA). One of the most commonly used monomers in dental applications is bisphenol A glycidyl methacrylate (BisGMA) [2]. The hydroxyl group and the stiff molecular structure of BisGMA are responsible for low polymerization shrinkage and good adhesion to enamel, yet these properties also result in high viscosity, as well as high water absorption.

The most commonly used diluent for BisGMA in dental applications is triethylene glycol dimethacrylate (TEGDMA), which is added to reduce the high viscosity of BisGMA in order to achieve a viscosity at which fillers can be incorporated [3]. BisGMA has been shown to have significantly higher stiffness than TEGDMA. The increased stiffness of BisGMA can hinder the mobility of the polymer chains during the curing process, leading to limited cross-linking and a decrease in conversion. These factors highlight the importance of selecting suitable monomers and optimizing their ratios to achieve the desired mechanical properties and degree of conversion in dental resins [4,5]. In comparison, it has been shown that a UDMA homopolymer has suitable hardness, flexural modulus, and flexural strength for dental applications [6,7]; however, a disadvantage is its high brittleness [8]. Based on these diverse material properties and their common use in dental applications, these materials were selected for inclusion in this study.

The mechanical properties of ceramic networks that serve as the inorganic phase for composites differ considerably. Often, good translucent esthetics are associated with poorer mechanical properties such as reduced flexural strength, while a highly stable ceramic network such as that of zirconia has a lower translucency and, thus, reduced esthetics [9].

Recently, the combination of ceramic networks and polymers, the so-called polymer-infiltrated ceramic network (PICN), has been the focus of research. A feldspar ceramic enriched with aluminum oxide is typically used. The advantage of this and of conventional composites is that they combine the mechanical properties, such as the modulus of elasticity, which is equivalent to dentin in polymer-based composites, and the strength of ceramics [10,11]. The advantage of a PICN over conventional composites is the enamel-like abrasion resistance; in addition, a PICN has improved fracture resistance compared to enamel [12]. The dental indication of a PICN is primarily single-tooth restorations.

A PICN already available for dental application is feldspar ceramic enriched with aluminum oxide, for which a flexural strength of 153.6 ± 10 MPa was determined in the biaxial (ball-on-ring) flexural strength test [13]. The aging of this PICN alumina-enriched feldspar ceramic was also investigated using thermocycling, followed by a flexural strength test, where a flexural strength of 141.6 MPa was recorded [14]. The mean Vickers hardness of this PICN was 254 HV, which is significantly higher than that of commercial composite resins (HV 75.6) [15,16]. The Vickers hardness is closer to that of enamel, which is 270–420 HV, than to that of dentin, which is 20–90 HV [17,18].

Another approach to the development of PICNs for dental applications could be the combination of a polymer and zinc oxide (ZnO). The advantage of ZnO is that it can be produced easily and cheaply using flame transport synthesis, compared to other manufacturing processes [19]. In addition, ZnO has an antibacterial effect, which, in contrast to conventional composite resins, could also make the material interesting for use in the oral cavity [20]. This is due to the antibacterial mechanism of ZnO, which causes the direct destruction of cell structures and membrane functions, as well as the generation of oxygen radicals for the oxidation of the biomacromolecules of the anaerobic Streptococcus mutans [21]. In addition, ZnO has not only antibacterial but also antiviral properties. Studies have shown that ZnO has an antiviral effect against herpes simplex virus types 1 and 2. The special surface of ZnO particles is of decisive importance here, as it induces an improved immune response in the body [22,23].

Furthermore, the addition of ZnO to established dental materials, such as glass ionomer cements, has been shown to enhance mechanical properties [24,25]. Therefore, replacing the ceramic part of a PICN with zinc oxide may add antiviral and antibacterial properties to the composite, and, combined with the mechanical properties of the polymer, it may improve the composite for dental applications [26].

However, the poor bonding of the polymer to the fillers, in this case ZnO, and the associated weaker mechanical properties [27] remain a challenge. Primers might be used to enhance the adhesion of the polymer to the sintered ceramic network, resulting in enhanced strength and increased durability. Primers containing MDP (10-methacryloyloxydecyl-dihydrogenphosphate), among others, have shown great potential and are well established in clinical and laboratory studies, for example, for the bond between the tooth structure and zirconium oxide [28]. Primers are bonding agents that improve the adhesive bond by enhancing the chemical bonding of the polymer [28,29]. Furthermore, adhesive ceramic restorations can be improved, as the contact area between the ceramic and polymer is completely wetted with the bonding agent. Thus, in addition to the enhanced chemical bonding, the wettability is increased, and the entrapment of air is prevented [30].

A lower contact angle between zirconia and resin improves the resin bond strength, and 10-MPD acidic functional monomers show a relative resistance to hydrolysis due to the long carbonyl chain [31]. In addition, the phosphate ester group in the adhesive monomers and zirconia oxides form direct chemical bonds. MDP has bifunctional ends with extended organic hydrophobic chain molecules. At one end, the hydrophilic phosphate ester groups adhere tightly to zirconia, while at the other end, the vinyl groups interact with the monomers in the resin cement [32]. Overall, it can be asserted that the MDP monomer enhances chemical bonding to oxide ceramics through a phosphate ester and a methacrylate group, exhibiting long-term durability in clinical settings [33]. This suggests that these attributes may similarly extend to tZnO properties, potentially enhancing bond strength and consequently improving the properties of the PICN.

For the clinical application of PICNs in dentistry, it is also important to test the water absorption, aging, and hydrolysis resistance. These properties are of central importance for the longevity of the dental material in the moist environment of the patient’s oral cavity. By measuring the roughness, conclusions can be drawn about the polishability and workability of the material.

In this study, a polymer-infiltrated ceramic network, with ZnO as the ceramic component, was produced. The properties investigated in this study were then examined depending on the polymer used for infiltration (BisGMA-TEGDMA or UDMA-TEGDMA). Furthermore, it was determined whether the use of a phosphate monomer containing a primer could also lead to an improvement in the bonding to the matrix and, thus, in the mechanical properties.

The null hypothesis of this study was that there is no statistically significant influence of the polymer type and that the application of an MDP-containing primer has no influence on properties such as the Vickers hardness, water absorption, roughness, and flexural strength of the PICN.

## 2. Materials and Methods

### 2.1. Specimen Preparation

**Figure 1:**
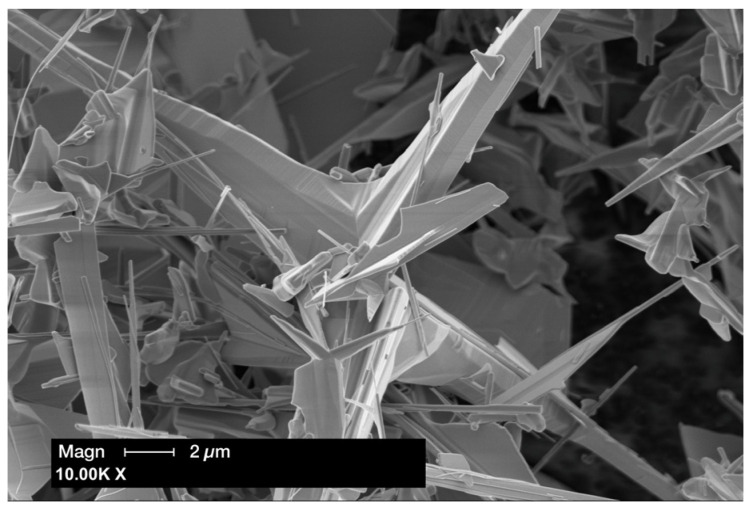
SEM image of the ZnO powder before sintering to form a network.

**Figure 2:**
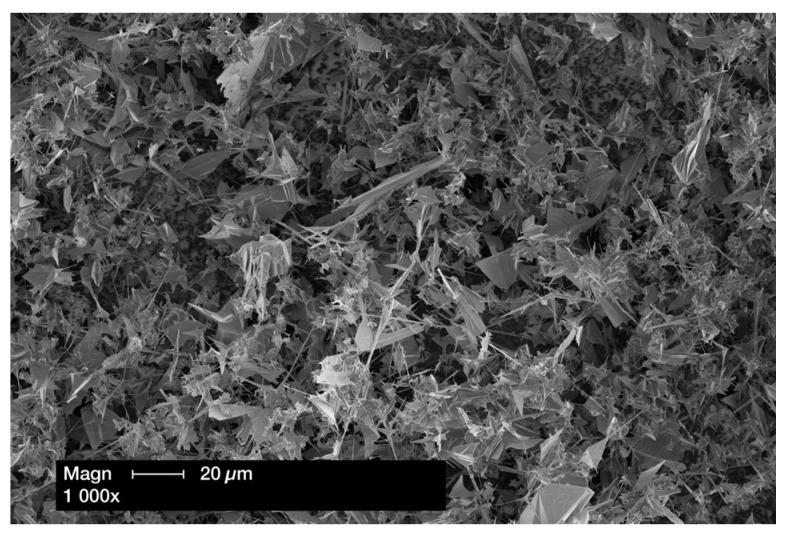
Exemplary cross-section SEM image of the ZnO network before polymer infiltration.

A total of 148 zinc oxide networks were fabricated using tetrapodal ZnO powder (tetrapodal zinc oxide; Phi-Stone AG, Kiel, Germany); see the SEM image in Figure 1. Tetrapodal-shaped ZnO microparticles consist of four hexagonally arranged projections in a wurtzite structure; in addition, the fabrication process produces the so-called sail structure of ZnO, as shown in Figure 1 [19,34]. The ZnO powder (0.3902 g) was compressed into a defined cylindrical geometry (a diameter of 12 mm and a height of 1.5 mm) using a metal mold and a press with a pressure of 10 kN to achieve a specimen density of 2.3 g/cm^3^ (PW; Paul-Otto Weber Laborpresstechnik, Remshalden, Germany), followed by sintering at 1150 °C for 5 h, after which no significant sintering shrinkage could be detected (Figure 2). The density of the resulting ZnO network led to a previously calculated ZnO concentration of 75 wt.% in the final PICN specimens. The 74 specimens, which were to be pre-treated with a primer containing the phosphate monomer MDP (Alloy Primer; Kurary Noritake Dental Inc., Okayama, Japan), were drizzled with 100 µL of the primer at room temperature under laboratory conditions before polymer infiltration. This corresponded to the calculated free volume of the ZnO networks.

The polymer BisGMA-TEGDMA (bisphenol A-glycidil methacrylate; triethylene glycol dimethacrylate; Sigma Aldrich, St. Louis, MO, USA) was weighted at a ratio of 60/40 wt.%, and TEGDMA-UDMA (urethane acrylate methacrylate resin; Sigma Aldrich) was weighted at a ratio of 40/60 wt.%. For BisGMA-TEGDMA, the photoinitiators camphorquinone (0.25 wt.%; Sigma Aldrich) and ethyl-4-dimethylaminobenzoate (0.25 wt.%; Sigma Aldrich) were mixed; for the UDMA-TEGDMA mixture, the photoinitiators camphorquinone (0.7 wt.%) and 2-(dimethylamino)-ethyl methacrylate (0.3 wt.%; Sigma Aldrich) were used. After mixing for 30 min using a magnetic stirrer in a dark fume hood, the polymers were stored in a vacuum chamber at 10 hPa for 30 min to avoid air entrapment. This specimen preparation and the polymer ratios suitable for the study were determined in a pilot study.

Both the pre-treated ceramic networks (*n* = 74) and the ceramic networks without the primer containing MDP (*n* = 74) were immersed in the corresponding viscous polymers and then stored for 30 min in a dark vacuum chamber to completely infiltrate the specimens. The specimens were then light-cured from both sides for 80 s at a distance of 4 mm (Radii-cal LED curing light; SDI, Victoria, Australia-Bayswater), with a tip size of 7 mm and a light output of 1200 W/cm^2^.

For comparison, *n* = 74 pure polymer specimens were each prepared from UDMA-TEGDMA (*n* = 37) and BisGMA-TEGDMA (*n* = 37). This was carried out by stirring the pure polymer for 30 min and then storing it for 30 min at 10 hPa in a vacuum chamber to exclude air inclusions. The specimens were finally poured into silicone molds, with a diameter of 12 mm, and light-cured from all sides for 80 s.

**Figure 3:**
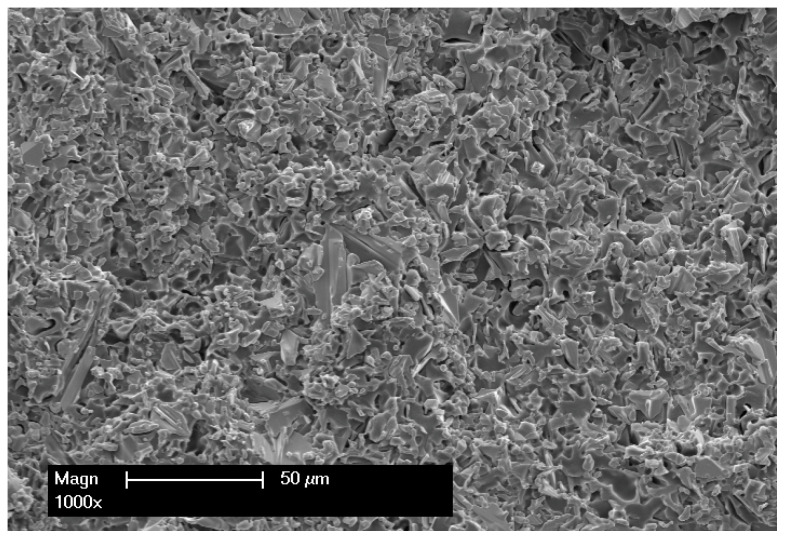
Exemplary cross-section SEM image of the PICN network after polymer infiltration.

Standardized test specimens were produced in the form of discs, with a diameter of 12 mm. Finally, all specimens were ground to a height of 1.2 mm using 600-grit silicon carbide paper and a polishing machine (EcoMet 250 Pro; Buehler, Lake Bluff, IL, USA). To ensure homogeneous infiltration in the individual specimens, cross-section SEM images of the PICN networks were taken in a preliminary study after the infiltration process. These images demonstrated homogeneous infiltration of the tZnO network with the corresponding polymer (Figure 3). In addition, the free volume of the specimens was 0.08 mm^3^, as calculated using a ZnO powder density of 2.3 g/cm^3^, which was used for specimen preparation. By weighing the specimens, the mean percentage infiltration rate of the free volume in the specimens by the polymers used was determined. The results showed an infiltration rate of 99.5% for the Z-UT-P specimens, 99.3% for the Z-UT specimens, 96.7% for the Z-BT-P specimens, and 98.5% for the Z-BT specimens.

The codes of the 6 groups containing 37 specimens each are described in Table 1.

### 2.2. Experiments

#### 2.2.1. Biaxial Flexural Strength with Thermocycling

A total of 20 specimens per group were used for the biaxial flexural test. Half of these randomized specimens (*n* = 10) were subjected to the biaxial flexural test without prior storage, and the other half (*n* = 10) of the randomized specimens were first stored for 150 days in a water bath with additional thermocycling (HAAKE W15; Mechatronik GmbH, Feldkirchen, Germany) before the flexural strength test was performed.

For water storage with additional thermocycling, the specimens were stored in a heat bath (Precision GP02; Thermo, Fisher Scientific, Newington, CT, USA) at 37 °C for 150 days. Within these 150 days, a total of 37,500 thermal cycles between 5 °C and 55 °C were performed. The specimens were kept in the baths for 30 s per cycle [35].

**Figure 4:**
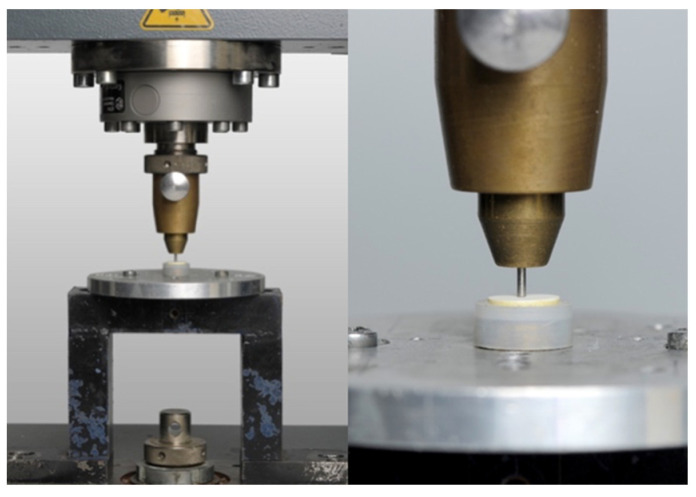
PICN specimen placed on three steel balls; the piston applies pressure to the specimen to determine the flexural strength (2-column fitting image).

The biaxial flexural strength test was performed on a universal testing machine (Zwick Z010/24; Zwick, Ulm, Germany) (Figure 4). The prefabricated specimens were placed on three spheres, each with a diameter of 3.2 mm, which were placed on a circle (radius of 5 mm) at an angle of 120° [36]. A quasi-static force was applied and increased by a cross-head speed of 0.5 mm/min until the specimen broke by loading it with a cylindrical punch in the center of the test. Due to the central loading, edge effects could be excluded [37]. Both the fracture load and the biaxial flexural strength could be determined with the following equation:

S = (−0.2387 × P × (X − Y))/d^2^

where S is the biaxial flexural strength (MPa); P is the fracture load (N); and d is the specimen disk thickness at the fracture origin (mm). X and Y were determined as follows:

X: (1 + ν) × [〖ln(r_2/r_3)〗^2^ + [(1 − ν)/2] × (r_2/r_3)^2^

Y: (1 + ν) × [1 + ln〖(r_1/r_3)^2^〗] + (1 + ν) × (r_1/r_3)^2^

where ν is Poisson’s ratio (0.25), r_1 is the radius of the support circle, r_2 is the radius of the load area, and r_3 is the radius of the specimen.

#### 2.2.2. Vickers Hardness Test

The Vickers hardness test was carried out according to DIN EN ISO 6507 [38] using a hardness tester (Zwick 3212; Zwick, Ulm, Germany) with *n* = 8 specimens. This test was conducted to provide a comparative value to other PICNs, such as an alumina-enriched feldspar ceramic, in which the Vickers hardness in particular was tested [16].

In this test, a pyramid-shaped indenter with a dihedral angle of 136° was pressed onto the test specimens for 20 s [13]. A load of 0.5 kg (HV 0.5) was used for the BisGMA-TEGDMA specimens, 0.2 kg (HV 0.2) for the UDMA-TEGDMA specimens, and 1 kg (HV 1) for the remaining ZnO specimens. The test was carried out at room temperature. With the help of the resulting diagonal lengths of the indentation, which were determined with a microscope, the Vickers hardness could be calculated:

$$\mathrm{HV}\approx 0.1891*F/d^{2}$$

- HV = Vickers hardness.
- F = Force in Newton.
- d = Lengths of the diagonals in mm.

#### 2.2.3. Roughness Measurement

The average roughness R_a_ (in µm) was determined on 3 specimens using a laser scanning microscope (VK-X 100 series; Keyence Corporation, Osaka, Japan). Here, a 0.00059 cm^2^ area of the specimen was examined with a laser wavelength of 658 nm and a 50 × magnification, whereby an average of 20 equidistant line scans was determined. For the measurement before fine polishing, the specimens were polished to 600 grit with polishing wheels. After the first measurement, the test specimens were polished in various steps to a 1 µm grain size, as shown in Table 2. For the last two polishing steps, a specific diamond suspension (MetaDI Supreme; Buehler, Lake Bluff, IL, USA) was used.

#### 2.2.4. Water Absorption

To evaluate water absorption, 6 specimens were preconditioned and dried at 37 °C for 24 h in a drying cabinet. The water absorption was measured gravimetrically with an accuracy of ±0.1 mg on a fine balance at room temperature. The specimens were stored in a water bath at 37 °C. Due to the density of the PICN being higher than that of the pure polymer specimens, the relative increase in water absorption was calculated:

$$x=100*\left(a-b\right)/b$$

- *x* = relative water uptake at time *x* (in %).
- *a* = measurement at time *x* (in grams).
- *b* = initial value (in grams).

The measurement was taken at the following time intervals: 5–40 min (every 5 min), 1–6 h (once per hour), 24 h, 34 h, 48 h, 58 h, 3 d, 5 d, 7 d, 12 d, and 19 d.

#### 2.2.5. Statistical Analysis

For a statistical evaluation, SPSS 30 software was used. Here, the Shapiro–Wilk test was used to determine the normal distribution for both the Vickers hardness test and the biaxial flexural strength test. As the data were not normally distributed, the Kruskal–Wallis test was used for a statistical evaluation, and the Mann–Whitney test, followed by Bonferroni–Holm correction, was used for pairwise comparisons. Descriptive statistics were used for the roughness measurements.

For the statistical analysis of the water absorption tests after 456 h, the normal distribution was also tested using the Shapiro–Wilk test, showing *p* > 0.05 for all groups. The homogeneity of variances was confirmed using Levene’s test (*p* > 0.05). Therefore, a one-way analysis of variance (one-way ANOVA) was performed with the post-hoc Tukey test specified.

## 3. Results

### 3.1. Flexural Strength Before and After Thermocycling

The medians, means, and standard deviations of the flexural strength measured in the piston-on-three-balls test, before and after the thermocycling process, are shown in Table 3.

In comparison to the PICN, the pure polymer specimens showed a statistically significant higher flexural strength (*p* ≤ 0.05). Additionally, the different material properties of the polymers used resulted in significant differences (*p* ≤ 0.05). All BisGMA-TEGDMA-containing groups showed a higher flexural strength (Z-BT 56.2 MPa) than the corresponding UDMA-TEGDMA-containing groups (Z-UT 50.0 MPa).

In addition, the statistical evaluation showed that the flexural strength of the groups with a ZnO network pre-treated with a primer containing MDP was significantly compared to that of the groups without a primed ZnO network.

**Figure 5:**
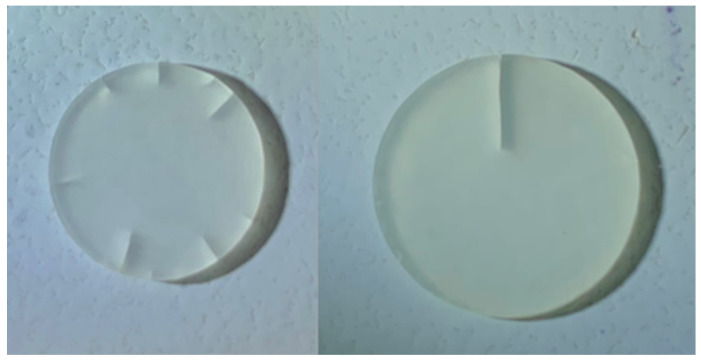
Different hydrolytic effects on BisGMA-TEGDMA specimens after 150 days of water storage with thermocycling.

The aging of the specimens with thermocycling resulted in a significantly reduced flexural strength. The highest median flexural strength was shown by the pure polymer BisGMA-TEGDMA specimens (123.1 MPa) before aging. However, it was found that BisGMA was already broken in different configurations, which was due to the manual production and thus, there were minimal differences between the specimens after aging, and its structure was affected by water storage (Figure 5), which drastically reduced the flexural strength compared to that of the unaged specimens.

### 3.2. Vickers Hardness Test

In Table 4, the medians, means, and standard deviations of the Vickers hardness are shown. According to the Wilcoxon–Mann–Whitney test, the measurement results and material properties of the specimens were significantly affected by each parameter, including the polymer, primer, and ZnO network. It should be noted that the Vickers hardness of the materials used varied greatly due to the different loads required for the measurement. The Vickers hardness ranged from 18.4 HV 0.5 (BT) for the pure polymer to 71.5 HV 1 (Z-BT) in the median. Therefore, the BisGMA-TEGDMA groups showed a higher Vickers hardness than the UDMA-TEGDMA groups. An exception is the pure polymer group, where the UDMA-TEGDMA group had a higher Vickers hardness than the BisGMA-TEGDMA group.

In general, the ZnO specimens had a higher Vickers hardness than the polymer specimens. The PICN pre-treated with an MDP-containing primer resulted in a statistically significant decrease in hardness compared to the PICN without primer application.

### 3.3. Roughness Measurement

The mean value and standard deviation of the mean roughness average (R_a_) and the total height of profile (R_t_) in micrometers before and after polishing are given in Table 5. Descriptive statistics were compiled for an analysis of the data.

The PICN specimens showed a higher roughness than the pure polymer specimens. Compared to the not-primed PICN, the PICN pre-treated with an MDP-containing primer demonstrated a reduced roughness. Clear differences between the material properties of UDMA-TEGDMA and BisGMA-TEGDMA were found.

**Figure 6:**
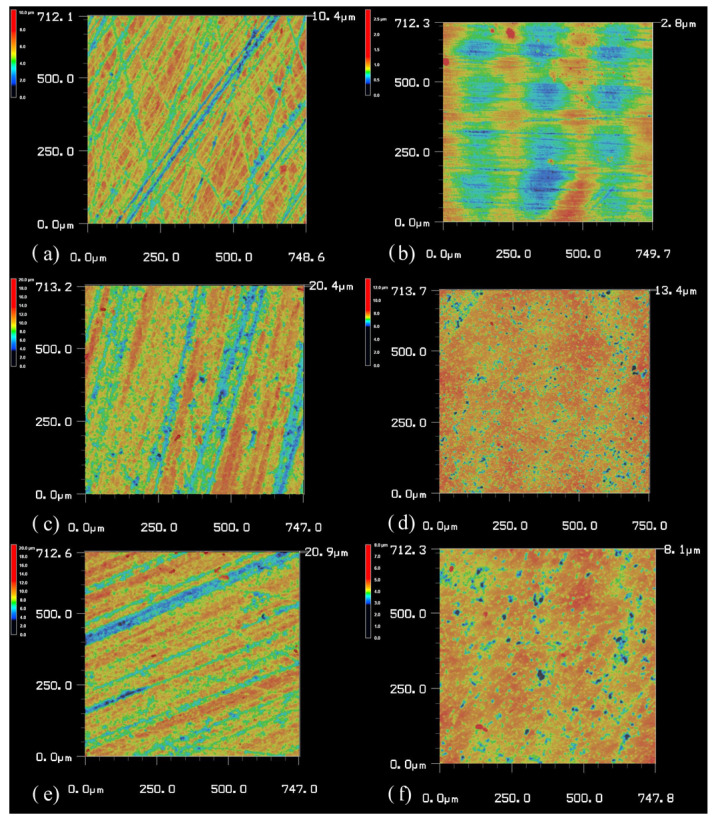
Topography images of different surface roughnesses obtained from confocal laser scanning microscopy of the BisGMA-TEGDMA specimens before and after polishing, marked with lowercase letters: (**a**) BT before; (**b**) BT after; (**c**) Z-BT before; (**d**) Z-BT after; (**e**) Z-BT-P before; (**f**) Z-BT-P after.

**Figure 7:**
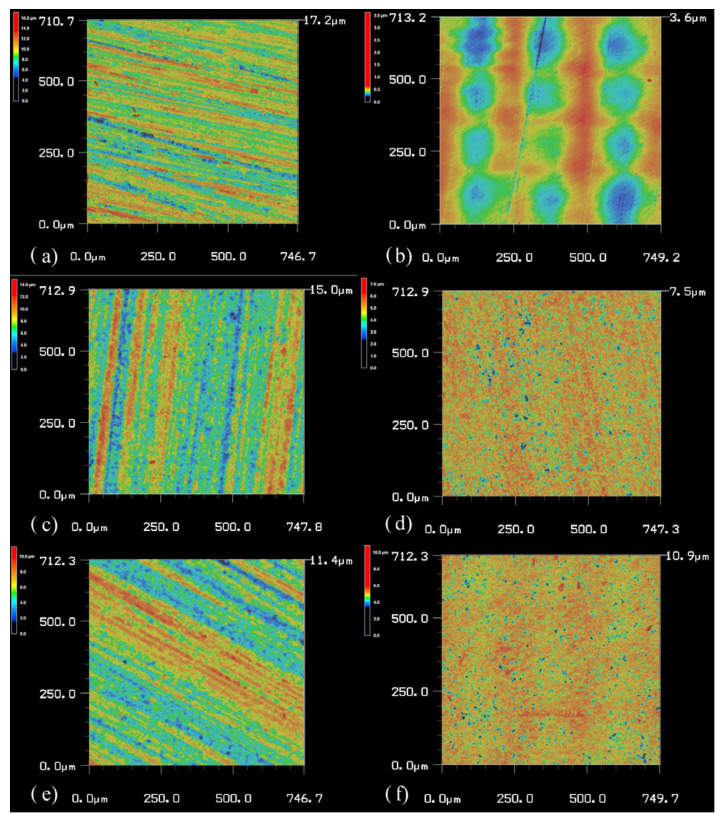
Topography images of different surface roughnesses obtained from confocal laser scanning microscopy of the UDMA-TEGDMA specimens before and after polishing, marked with lowercase letters: (**a**) UT before; (**b**) UT after; (**c**) Z-UT before; (**d**) Z-UT after; (**e**) Z-UT-P before; (**f**) Z-UT-P after.

The surface roughness results are illustrated in Figure 6 and Figure 7, which show topography images captured using confocal laser scanning microscopy.

### 3.4. Water Absorption

**Figure 8:**
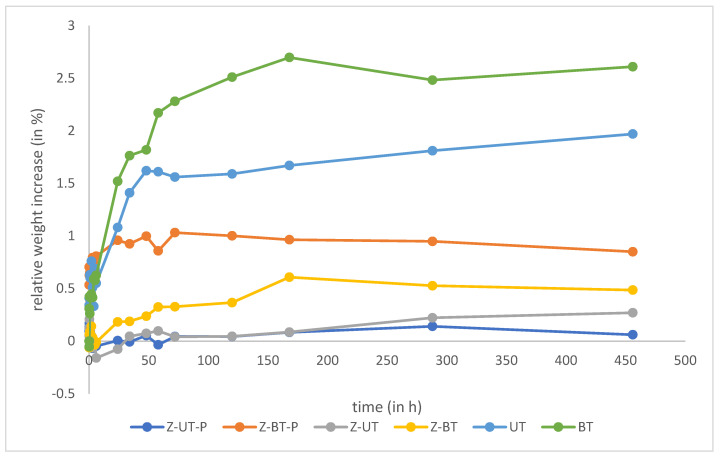
Relative water absorption in all test groups from 0 to 456 h (19 days).

The relative water absorption in percentage from 0 to 456 h (19 days) is graphically shown in Figure 8, the mean value and the standard deviation of the relative weight gain in percent are also shown in Table 6. The statistical analysis revealed that the water absorption of the pure polymer specimens differed statistically significantly from all other specimen groups, showing a higher water absorption in comparison. However, the increased water absorption of the BisGMA-TEGDMA groups compared to the UDMA-TEGDMA-containing groups showed no significance (*p* > 0.05) in the statistical analysis. Generally, the highest water absorption was observed in the pure BisGMA-TEGDMA (*p* < 0.05) specimens.

## 4. Discussion

### 4.1. Biaxial Flexural Strength

The lower stability of the UDMA-TEGDMA pre-treated PICN in the biaxial flexural strength test compared to the BisGMA-TEGDMA groups can be explained by the different material properties of the polymers [39]. The mechanical properties of the polymers are linked to their different monomer structures [40]. In Bis-GMA-containing groups, the hydroxyl groups and rigid molecular structure contribute to low stiffness but high viscosity and water absorption. In contrast, the NH---N and NH---O hydrogen bonds in the UDMA system exhibit lower strength than the O-H---O hydrogen bonds that predominate in the Bis-GMA system. These factors may explain the results of the biaxial flexural strength test, among others [8].

In flexural strength testing, thermocycling also makes a significant difference to the specimens; this is explained by the hydrolytic effects on the different polymers, which are susceptible to different degrees of hydrolysis depending on their chemical composition [31,41,42]. The effect of the hydrolytic decomposition of the different polymers was especially evident in the pure BisGMA-TEGDMA specimens. After 150 days of water storage with thermocycling, these specimens showed clear damage, which could be explained by the hydrolysis effect (Figure 5) [42,43]. The UDMA-TEGDMA specimens were less affected by hydrolytic effects, resulting in the pure UDMA-TEGDMA specimens achieving higher biaxial flexural strength values than the aged BisGMA-TEGDMA specimens.

Nevertheless, a significant deterioration in the results of the flexural strength test was observed in each individual test group after 150 days of water storage with thermocycling. Consequently, adjusting the polymer composition or using a customized primer based on this knowledge of hydrolysis susceptibility could lead to an improvement in the mechanical properties of the material.

Furthermore, the use of a ZnO network in the flexural strength test resulted in a flexural strength lower than that of the pure polymers. This could be related to the structure of the ZnO network. The ZnO network used has very thin structures compared to other polymer-infiltrated ceramic networks [34,44]. This and the so-called “sail formation” that results from sintering mean that the network is generally not very stable, both in the flexural strength test and in other tests [34]. The flexural strength was significantly lower than that of other polymer-infiltrated ceramic networks, such as feldspar ceramics enriched with aluminum oxide, which have been found to have a flexural strength of 153.6 MPa in the biaxial (ball-on-ring) flexural strength test [13]. The higher strength values of other PICN materials, such as feldspar ceramics enriched with aluminum oxide, can be attributed to their structure. These materials have significantly thicker structures within the ceramic matrix and a three-dimensional, interconnected dual-network structure. This network structure was also found in the ZnO-PICN used in the present study [45].

The MDP monomer-containing primer also had a significant influence on the flexural strength. One of the reasons for this is that the primer improves the adhesion of the polymer to the ZnO network matrix by reducing the contact angle between the ceramic matrix and the resin [31]. In addition, this type of primer improves the quality of the bond with ceramic restorations by completely wetting the surface of the ceramic network. This improved wettability has a positive effect on the chemical bond and prevents the penetration of air. In addition, a phosphate monomer-containing primer chemically binds to both the ceramic and the organic part of the polymer, which leads to increased stability of the entire PICN [30,46,47]. This improved bonding between the polymer and the ZnO network means that the connections are less susceptible to failure under the stress caused by thermocycling. As a result, an increase in the stability of the PICN can be observed, as the flexural strength test showed. The clinically established MDP monomer-containing primer used in this study could be further investigated or exchanged in further studies in order to possibly enhance the already improved material properties.

The investigations carried out show that the flexural strength values of the PICNs produced in the present study are significantly reduced in comparison to those of not only the human tooth structure [48] but also other PICNs, such as feldspar ceramics enriched with aluminum oxide. The very thin structures of the ZnO network used make it unsuitable for dental applications in terms of flexural strength. Here, correspondingly adapted thicker structures could lead to an improvement in the flexural strength and thus to better suitability for dental applications.

A limitation of this investigation can be found in the production of the specimens. Due to the manual steps, such as weight determination, pressing, infiltration, and grinding, the manufactured specimens have a lower manufacturing precision compared to industrial production. Nevertheless, to examine the production quality and homogeneity, for example, in the infiltration, the specimens were tested in preliminary studies for their quality and homogeneity using SEM and by calculating the infiltration rate of the free volume within the specimens. Standardized automated production would improve the precision of specimen and material production. This approach could be implemented as soon as the optimal combination of materials has been determined.

### 4.2. Vickers Hardness

The Vickers hardness test showed a clear influence of the polymer on the test results. The variations in the Vickers hardness of the individual specimens were highlighted by the need to apply different load magnitudes (HV 0.2–HV 1) in order to achieve a measurable effect. The pure UDMA-TEGDMA specimens showed a higher Vickers hardness (33.1 HV 0.2) than the pure BisGMA-TEGDMA specimens (18.3 HV 0.5) [49]. This phenomenon can be explained by the more elastic behavior of the UDMA-TEGDMA specimens [50].

Due to the higher elasticity of the pure polymer specimens in general, their Vickers hardness values were significantly reduced compared to those of the PICN specimens with a ceramic ZnO network, whose elasticity is very limited [51]. This is due to the fact that ZnO additionally stabilizes the surface of the polymer network due to its stronger ceramic network [52]. For this reason, the PICNs had higher Vickers hardness values than the pure polymer specimens [53].

### 4.3. Roughness

The significant difference in roughness between the PICN specimens and pure polymer groups can be attributed to the weaker bond between the ceramic networks and the corresponding polymer [54], which results in the PICNs being less polishable than the pure polymer groups. This effect can be explained by the fact that the tZnO particles are torn out of the network during polishing. The fine structures of the ZnO network could also explain this effect, as the comparatively thin arms of the ZnO tetrapods easily fracture [34], which leads to significantly higher roughness values and, thus, lower polishability.

Nevertheless, the utilization of an MDP-containing primer can augment the adhesion between the ceramic network and the pristine polymer due to the chemical interactions between the MDP-infused primer and the ZnO network [28,31]. This enhancement in adhesion leads to a reduced occurrence of tZnO particle extraction from the specimens during the polishing process. The improved adhesion can be explained by the fact that the phosphate ester groups in the MDP-containing primer form direct chemical bonds with the ZnO network, similar to zirconia [33]. The lower occurrence of ZnO particle extraction and the resulting surface, where fewer whole particles are removed and usually only individual arms of the tetrapods break off, explain the lower roughness value and the resulting higher polishability of the PICN with the MDP-containing primer.

In this study, only the general polishing capability was tested using a diamond suspension. The extent to which chairside polishing can be performed with conventional dental polishers must be evaluated in future studies.

### 4.4. Water Absorption

The water absorption experiment showed that the PICN group had a lower absorption rate than the other groups, such as the BisGMA-TEGDMA specimens; which are known to absorb larger amounts of water, and can lead to hydrolytic effects [43,55]. As with other ceramic networks, such as feldspar ceramics or ZnO, it is possible to reduce water absorption by producing a PICN [56]. The reason behind this is that materials with hydrophilic monomers have a higher water absorption rate [57], while PICN specimens with a ZnO network have lower hydrophilicity and, thus, lower water absorption [58]. These hydrophilic properties led to the PICN groups absorbing less water than the pure polymer groups.

Unfortunately, in laboratory tests, the water absorption of resins shows only limited comparability to the conditions in the oral cavity. Studies have shown that the water absorption of composite resins depends on the storage temperature [59]. In this laboratory study, the specimens were stored at a constant temperature of 37 degrees Celsius. It should be noted that significant thermal cycling occurs during the course of the day due to food intake. In addition, the average temperature in the oral cavity varies depending on the location in the mouth and ethnic aspects. In general, the temperature in the oral cavity can average between 33 °C and 37 °C [60]. The pH value, which fluctuates throughout the day in the oral cavity, could also be investigated in further studies in order to take this factor into account in relation to the experiments carried out.

## 5. Conclusions

By taking the results and conditions of this study into account, the following conclusions can be drawn:

1. The PICN investigated in this study currently shows limitations for use in single-tooth restorations. This may be due to the fine structure of the ZnO network. A change in these structures would lead to an improvement in the mechanical properties, but this still needs to be investigated in further studies.
2. The use of a primer containing a phosphate monomer leads to an enhancement of the mechanical properties, as the bonding between the polymer and ceramic is enhanced. This finding is relevant for future studies aiming to investigate dental restorative materials with ZnO-based PICNs.

## Figures and Tables

**Table 1:** Codes for each group of specimens.

Group Code	Material	Polymer	Primer
Z-BT-P	PICN	BisGMA-TEGDMA	yes
Z-UT-P	PICN	UDMA-TEGDMA	yes
Z-BT	PICN	BisGMA-TEGDMA	no
Z-UT	PICN	UDMA-TEGDMA	no
BT	pure polymer	BisGMA-TEGDMA	no
UT	pure polymer	UDMA-TEGDMA	no

**Table 2:** Polishing steps of the specimens for the roughness measurement.

Step	Material for Polishing	Time (s)
1	600-grit silicon carbide paper	10
2	1000-grit silicon carbide paper	15
3	3 µm diamond suspension	45
4	1 µm diamond suspension	60

**Table 3:** Medians and means (standard deviations) of the biaxial flexural strength in [MPa] with and without aging for all groups. Statistically different medians (*p* ≤ 0.05) are indicated by different uppercase letters (within a column for the same material and primer) or lowercase letters (within a column for BT) and lowercase Greek letters (within a column for UT).

Group	Initial		150 Days	
	Median	Mean (Standard Deviation)	Median	Mean (Standard Deviation)
Z-BT-P	60.8 ^Ab^	63.6 (10.0)	55.8 ^Ba^	52.9 (5.2)
Z-UT-P	62.4 ^Aβ^	64.2 (9.3)	47.9 ^Bβ^	49.5 (4.2)
Z-BT	56.2 ^Ab^	55.5 (4.6)	42.8 ^Bb^	44.4 (6.7)
Z-UT	50.0 ^Aγ^	48.7 (5.0)	36.7 ^Bγ^	37.9 (3.9)
BT	123.1 ^Aa^	131.9 (25.9)	35.5 ^Bab^	51.6 (36.4)
UT	100.1 ^Aα^	98.5 (8.3)	73.7 ^Bα^	73.5 (8.3)

**Table 4:** Median in [HV 1; HV 0.5; HV 0.2] and means (standard deviation) of the Vickers hardness. Statistically different means (*p* ≤ 0.05) are indicated by different uppercase letters (for primer application) or lowercase letters (for the different polymers used).

Group	Unit	Median (HV)	Mean (Standard Deviation)
Z-BT-P	HV 1	62.2	60.2 ^Ba^ (6.0)
Z-UT-P	HV 1	52.3	49.3 ^Bb^ (5.1)
Z-BT	HV 1	71.5	71.3 ^Aa^ (1.7)
Z-UT	HV 1	60.2	60.3 ^Ab^ (2.4)
BT	HV 0.5	18.4	18.3 (0.7)
UT	HV 0.2	33.0	33.1 (0.9)

**Table 5:** Mean (standard deviation) R_a_ and R_t_ [µm] of each group.

Group	Initial (600 Grit)		After Polishing (1 µm)	
	R_a_	R_t_	R_a_	R_t_
Z-BT-P	0.67 (0.07)	5.6 (0.73)	0.22 (0.03)	2.88 (0.57)
Z-UT-P	0.81 (0.19)	6.38 (1.47)	0.25 (0.04)	3.15 (0.14)
Z-BT	0.76 (0.01)	6.82 (0.39)	0.34 (0.09)	4.25 (1.00)
Z-UT	1.01 (0.22)	9.60 (4.78)	0.45 (0.02)	4.28 (0.77)
BT	0.62 (0.05)	5.19 (0.10)	0.06 (0.06)	1.22 (1.50)
UT	0.75 (0.17)	6.54 (0.89)	0.02 (0.01)	0.33 (0.10)

**Table 6:** Mean (standard deviation) relative weight increase (in %) in all test groups after 456 h of water storage. Statistically significant differences (*p* ≤ 0.05) are indicated by different uppercase letters.

Group	Mean (Standard Deviation)
Z-BT-P	0.85 ^B^ (0.57)
Z-UT-P	0.06 ^C^ (0.40)
Z-BT	0.49 ^BC^ (0.25)
Z-UT	0.27 ^BC^ (0.18)
BT	2.61 ^A^ (0.45)
UT	1.97 ^A^ (0.71)

## Data Availability

The original contributions presented in the study are included in the article, further inquiries can be directed to the corresponding author.
